# Supplementary material for: Effective DDoS attack detection in software-defined vehicular networks using statistical flow analysis and machine learning
Source: PLoS One. 2024 Dec 18;19(12):e0314695. doi: 10.1371/journal.pone.0314695 (PMC11654938; doi:10.1371/journal.pone.0314695)
Supplement: Attachment — Submitted filename: revision 1 (3).docx [file pone.0314695.s001.docx]

**Point-wise Detailed Response to Editor and Reviewers’ comments**

Title: Effective DDoS Attack Detection in Software-Defined Vehicular Networks Using Statistical Flow Analysis and Machine Learning

Authors: Himanshi Babbar, Shalli Rani, Maha Driss

Dear Editors and Reviewers:

We are thankful to you for spending your valuable time for making a review and for constructing the comments on our manuscript. These comments are valuable and very helpful for revising and improving our paper. We have studied comments carefully and have made correction as marked in the revised manuscript. We have tried our best to address the mentioned comments to revise our manuscript in the hope that these revisions will meet your requirement. The following changes have been made in the manuscript as per the received comments.

*The changes made in the manuscript as per received comments have been highlighted in blue color.

**Reviewer #1:**

1. What are the potential limitations of using a small fraction of collected features for model evaluation, and how might this impact the generalizability of the results?

Response: Using a small fraction of collected features for model evaluation in detecting DDoS attacks in Software-Defined Vehicular Networks (SDVN) presents several potential limitations that can impact the generalizability of the results:

**a. Loss of Information:** By selecting only a subset of features, important information that could be crucial for distinguishing between different types of traffic (e.g., benign, reconnaissance, DoS, DDoS) might be omitted. This can lead to a decrease in the model's ability to accurately identify all types of malicious activities.

**b. Bias in Feature Selection:** The chosen subset of features might not represent the entire feature space adequately, leading to a biased model. This bias can result in a model that performs well on the training and validation datasets but poorly on unseen data, thereby reducing its generalizability.

**c. Underfitting Risk:** When a limited number of features are used, the model might not capture the complexity of the data, leading to underfitting. Underfitting occurs when the model is too simple to capture the underlying patterns in the data, resulting in poor performance across different datasets.

**d. Over-reliance on Selected Features:** The model may become overly reliant on the selected features, which could be specific to the dataset used for training and evaluation. This over-reliance can cause the model to perform inadequately when exposed to different datasets with varying distributions of features.

**e. Reduced Robustness:** A model trained on a limited set of features may lack robustness to variations and anomalies in real-world traffic. This can make the model less effective in adapting to new types of attacks or changes in traffic patterns that were not represented in the training data.

2. By addressing these considerations, the model can be made more resilient and effective in real-world applications, thereby enhancing its capability to generalize beyond the training data.

Response: By addressing these considerations, the model can be made more resilient and effective in real-world applications, thereby enhancing its capability to generalize beyond the training data:

**a. Comprehensive Feature Selection:** Ensure thorough feature selection by evaluating the importance of each feature using methods like recursive feature elimination (RFE) or feature importance from tree-based algorithms. This helps in retaining the most relevant features and reducing the risk of excluding critical information.

**b. Cross-Validation Techniques:** Implement robust cross-validation methods, such as k-fold cross-validation, to assess model performance across multiple subsets of the data. This approach ensures that the model's performance is consistent and not dependent on a specific subset, enhancing its generalizability.

**c. Dimensionality Reduction:** Apply dimensionality reduction techniques such as Principal Component Analysis (PCA) to reduce the feature space while retaining essential information. This helps in capturing complex relationships within the data, improving the model's ability to detect sophisticated attack patterns.

**d. Diverse Dataset Evaluation:** Validate the model using multiple datasets with varying characteristics and distributions. This ensures that the model does not overfit to a single dataset and performs well in different real-world scenarios, improving its robustness and adaptability.

**e. Data Augmentation:** Use data augmentation techniques to create synthetic data that represents potential real-world scenarios. This helps in training the model to recognize a wider variety of attack patterns and normal behaviors, enhancing its generalization capability.

**f. Regularization Methods:** Incorporate regularization techniques such as L1 (Lasso) or L2 (Ridge) regularization to prevent overfitting. Regularization helps in keeping the model simpler and more generalizable by adding a penalty for larger coefficients.

By integrating these strategies, the proposed Machine Learning (ML) model for detecting DDoS attacks in Software-Defined Vehicular Networks (SDVN) can achieve greater resilience, adaptability, and effectiveness. This enhances the model's capability to generalize beyond the training data, making it more reliable and practical for real-world applications in vehicular networks.

3. How does the proposed approach ensure that the differentiation between reconnaissance, DoS, and DDoS traffic is accurate and not influenced by the issues of unbalanced datasets?
Response: The proposed approach ensures accurate differentiation between reconnaissance, Denial of Service (DoS), and Distributed Denial of Service (DDoS) traffic by addressing the issues of unbalanced datasets and overfitting through several key strategies:

a. By measuring statistical flow characteristics and computing entropy, the approach captures distinct patterns and anomalies in network traffic. This helps in distinguishing between different types of malicious activities based on their unique statistical signatures.

b. The deployment of machine learning (ML) algorithms specifically designed for intrusion detection in Software-Defined Networking (SDN) environments enhances the model's ability to learn and recognize complex attack patterns. These algorithms are trained to differentiate between reconnaissance, DoS, and DDoS attacks by learning from labeled examples.

c. The approach explicitly tackles the issue of unbalanced datasets, which is common in cybersecurity datasets where benign traffic vastly outnumbers malicious traffic. Techniques such as oversampling the minority class, undersampling the majority class, or using synthetic data generation (e.g., SMOTE) can be employed to balance the dataset, ensuring that the ML model is exposed to sufficient examples of all types of traffic.

d. By using techniques such as cross-validation and regularization, the approach mitigates the risk of overfitting, where the model would perform well on training data but poorly on unseen data. This ensures that the model generalizes well and accurately classifies new, unseen traffic.

e. By using a small fraction of collected features and conducting a comparative study to identify the optimal sample size, the approach ensures that the most informative features are selected. This reduces noise and irrelevant information, improving the model's performance and accuracy in distinguishing different types of attacks.

f. The comparative study to ascertain the impact of different attributes on threshold performance helps in fine-tuning the model. This involves experimenting with various features and their combinations to find the optimal set that maximizes accuracy and detection capabilities.

g. The experimental evaluation using the BoT-IoT dataset, which contains diverse and realistic examples of IoT-related attacks, provides a robust testing ground for the model. The results showing high Precision, F1-score, Accuracy, and Recall for the Random Forest classifier indicate the effectiveness of the approach in accurately differentiating between different types of malicious traffic.

4. Why were $K$-nearest Neighbor, Random Forest, and Logistic Regression specifically chosen as the classifiers for this study, and what are the advantages and disadvantages of each in the context of SDN Intrusion Detection for IoT environments?

Response: The choice of \(K\)-nearest Neighbor (KNN), Random Forest, and Logistic Regression classifiers for this study in the context of SDN Intrusion Detection for IoT environments is based on their distinct characteristics and strengths that make them suitable for different aspects of intrusion detection. Here are the reasons for selecting these classifiers, along with their respective advantages and disadvantages:

(K\)-nearest Neighbor (KNN)

Reasons for Selection:

- KNN is easy to understand and implement. It is a straightforward algorithm that classifies a data point based on the majority class of its \(K\) nearest neighbors. It makes no explicit assumptions about the distribution of data, which is beneficial when dealing with complex and non-linear relationships in IoT traffic.

Advantages:

- Performs well on small datasets where the computational cost is manageable.

- Can handle multi-class classification without any modification.

- KNN is a lazy learner, meaning it requires no explicit training phase, making it fast to deploy.

Disadvantages:

- High computational cost during prediction as it involves calculating the distance between the query point and all points in the dataset.

- Requires storing the entire dataset, which can be impractical for large datasets.

- Performance can be degraded by irrelevant or redundant features, necessitating careful feature selection and normalization.

**Random Forest**

Reasons for Selection:

- Random Forest is an ensemble method that combines multiple decision trees to improve accuracy and control overfitting. It provides insights into feature importance, which is valuable for understanding which features are most indicative of an attack.

Advantages:

- Generally provides high accuracy and robustness to overfitting due to averaging multiple trees.

- Can handle both classification and regression tasks and works well with high-dimensional data.

- The ensemble nature reduces the risk of overfitting compared to individual decision trees.

Disadvantages:

- More complex and computationally intensive than single decision trees, particularly during the training phase.

- While individual trees are interpretable, the overall Random Forest model is less transparent.

- Can be slower to train, especially with large datasets and a high number of trees.

Logistic Regression

Reasons for Selection:

- Logistic Regression is a simple yet effective linear model that provides clear probabilistic interpretations of the classification results.

- Often used as a baseline classifier due to its simplicity and ease of implementation.

Advantages:

- Computationally efficient and fast to train, making it suitable for real-time applications.

- Provides coefficients that are easily interpretable, indicating the strength and direction of the relationship between features and the outcome.

- Effective for binary classification tasks, which are common in intrusion detection.

Disadvantages:

- Assumes a linear relationship between the features and the log odds of the outcome, which may not capture complex, non-linear patterns in IoT traffic.

- May perform poorly if the underlying data relationships are non-linear and complex.

- Can be sensitive to outliers, which can skew the model's performance.

5. How does the proposed approach measure statistical flow and compute entropy, and how are these measurements integrated into the Machine Learning algorithms for intrusion detection?
Response: **Statistical flow measurements** involve collecting and analyzing various metrics that describe the behavior of network traffic. Common metrics include:

Packet Count: Number of packets transmitted over a period.

Byte Count: Amount of data (in bytes) transmitted over a period.

Flow Duration: The time duration of a flow from the first to the last packet.

Packet Inter-arrival Time: Time intervals between consecutive packets.

Flow Rate: The rate at which packets/bytes are transmitted.

These metrics help in understanding the patterns and anomalies in network traffic. For instance, a sudden spike in packet count or flow rate can indicate a potential attack.

**Computing Entropy**

Entropy is a measure of randomness or unpredictability in a dataset. In the context of network traffic, entropy can help in identifying irregular patterns that deviate from normal behavior. The steps to compute entropy are as follows:

Categorize Traffic: Divide the network traffic into different categories based on features like source/destination IP addresses, port numbers, and protocols.

Probability Distribution: Calculate the probability distribution of these categories. For example, the probability P_i of each unique source IP address in the traffic.

Entropy Calculation: Use the probability distribution to compute the entropy using the formula:

H=−i∑Pilog2(Pi)

Here, H is the entropy, and P_i is the probability of the i-th category.

High entropy indicates a high level of randomness (potentially normal traffic), while low entropy can indicate more predictable and potentially malicious traffic patterns.

**Integration into Machine Learning Algorithms**

The statistical flow measurements and entropy values are integrated into ML algorithms as features. Here’s how this integration typically works:

Feature Extraction: Extract statistical flow metrics and entropy values for each traffic flow. These become part of the feature set used to represent the data.

Feature Vector Construction: Construct feature vectors for each traffic instance, combining traditional network features (like IP addresses and port numbers) with statistical metrics and entropy values.

Model Training: Use the constructed feature vectors to train ML models. The ML algorithms learn patterns in the feature space that differentiate normal traffic from various types of malicious traffic (reconnaissance, DoS, and DDoS).

Model Evaluation: Evaluate the models using metrics like accuracy, precision, recall, and F1-score to ensure they can accurately detect and classify different types of attacks.

6. What criteria were used to determine the best sample size for the dataset, and how was the balance between sample size and model performance evaluated?

Response: Determining the best sample size for the dataset and evaluating the balance between sample size and model performance involves several critical steps and criteria. Here's a detailed explanation of the process:

**Criteria for Determining the Best Sample Size**

a. Evaluate key performance metrics such as accuracy, precision, recall, and F1-score. These metrics provide a comprehensive view of the model's ability to correctly identify different types of traffic, including benign and malicious traffic (reconnaissance, DoS, DDoS).

b. Consider the training time and computational resources required. Larger sample sizes typically improve model performance but also increase the time and resources needed for training.

c. Monitor for signs of overfitting (where the model performs well on training data but poorly on validation/testing data) and underfitting (where the model performs poorly on both training and validation/testing data). The goal is to find a sample size that minimizes both.

d. Ensure that the sample size is large enough to achieve statistically significant results. Small sample sizes may lead to high variance in performance metrics and unreliable conclusions.

e. Ensure that the sample size captures the diversity and represents the full spectrum of network traffic behaviors, including different types of attacks and normal traffic patterns.

7. What are the implications of the Random Forest classifier achieving the highest performance metrics, and how might this influence the choice of ML algorithms for future studies in similar contexts?
Response: The Random Forest classifier achieving the highest performance metrics in this study has several important implications and can significantly influence the choice of machine learning (ML) algorithms for future studies in similar contexts. Here are the key implications and potential influences:

**Implications**

The high performance of the Random Forest classifier suggests that it is robust and reliable for detecting DDoS attacks in Software-Defined Vehicular Networks (SDVN). This robustness comes from its ensemble nature, which combines multiple decision trees to reduce variance and avoid overfitting.

Random Forest inherently provides a measure of feature importance, which helps in understanding which features are most significant for detecting anomalies and attacks. This insight can be valuable for refining feature selection and improving model performance.

The ability of Random Forest to handle imbalanced datasets effectively is crucial in intrusion detection, where malicious traffic is often much less frequent than normal traffic. The algorithm's ability to maintain high precision and recall indicates its strength in dealing with such imbalances.

The success of Random Forest in this study demonstrates its scalability and applicability to large datasets. It can handle a high-dimensional feature space and large amounts of data without significant performance degradation.

8. How does the proposed approach handle the dynamic and evolving nature of IoT environments, particularly in terms of updating the intrusion detection models and dealing with new types of attacks?

Response: The proposed approach addresses the dynamic and evolving nature of IoT environments by implementing adaptive strategies that ensure intrusion detection models remain effective against emerging threats and changing network conditions. Key to this approach is the utilization of machine learning techniques that support continuous learning and adaptation. **Firstly**, the model is designed to incorporate streaming data, allowing it to dynamically update its understanding of normal and anomalous traffic behaviors in real-time. This is crucial in IoT environments where traffic patterns can shift rapidly due to new devices, applications, or attack methodologies. **Secondly**, the approach includes mechanisms for regular model retraining using the latest data. By periodically refreshing the model with up-to-date information, it can adapt to new types of attacks and evolving tactics used by adversaries. **Thirdly**, the system employs anomaly detection algorithms that can detect deviations from established behavioral baselines, thereby identifying novel attack patterns that may not have been encountered previously. Additionally, the approach leverages threat intelligence feeds and collaborative security platforms to stay informed about emerging threats and vulnerabilities specific to IoT environments. By combining these elements—real-time data integration, periodic model updates, anomaly detection, and threat intelligence—the approach ensures that intrusion detection capabilities remain robust and responsive in safeguarding IoT networks against both known and unforeseen security risks. This proactive and adaptive methodology enhances the resilience of IoT security frameworks, mitigates potential risks, and supports the safe deployment and operation of connected devices in dynamic IoT ecosystems.

**Reviewer #2:** 
1. Please discuss how you will differentiate DDoS attack packet flow and legitimate packet flow?

Response: To differentiate DDoS attack packet flows from legitimate packet flows effectively, several sophisticated techniques and strategies can be employed. Firstly, monitoring traffic volume and rates is crucial; DDoS attacks typically generate a significantly higher volume of packets per second compared to normal traffic patterns. Analyzing these metrics allows for the detection of sudden spikes or anomalies that may indicate an ongoing attack. Secondly, statistical analysis plays a pivotal role by examining packet inter-arrival times and entropy. DDoS attacks often exhibit irregular patterns and lower entropy due to the repetitive nature of attack packets, which contrasts with the more diverse and predictable patterns of legitimate traffic. Thirdly, leveraging machine learning models, such as Random Forest or Support Vector Machines (SVMs), trained on historical data helps classify traffic based on learned patterns of normal versus attack behavior. These models can discern subtle deviations from established norms, enhancing the ability to detect and respond to emerging threats in real-time. Additionally, protocol and payload inspection techniques scrutinize network protocols and packet contents for anomalies indicative of attack signatures or malicious intent, providing deeper insights into the nature of traffic anomalies. By integrating these methods within a comprehensive detection framework, organizations can effectively differentiate DDoS attack packet flows from legitimate traffic, thereby fortifying their defenses and ensuring the resilience of their networks against evolving cyber threats.

2. Add a major limitation of the model in conclusion.
Response: Each machine learning algorithm, including K-Nearest Neighbors (KNN), Logistic Regression, and Random Forest, has its own set of limitations, which are important to consider when selecting an appropriate algorithm for a specific task. Here are the limitations associated with each of these algorithms:

**K-Nearest Neighbors (KNN)**

Computational Complexity: KNN can be computationally expensive, especially when dealing with large datasets or high-dimensional data. The algorithm needs to calculate distances between the query instance and all training instances, which can become impractical in large-scale applications.

Memory Usage: KNN requires storing all training data, as it makes predictions based on the nearest neighbors. This can lead to high memory usage, particularly with large datasets.

Sensitive to Irrelevant Features: KNN is sensitive to irrelevant or redundant features because it calculates distances using all available features. Noisy or irrelevant features can significantly impact its performance.

Need for Optimal K-Value: The choice of the parameter k (number of neighbors) in KNN is critical. A small k may lead to noise sensitivity, while a large k may cause over-smoothing, potentially missing local patterns in the data.

Imbalanced Data: KNN can perform poorly with imbalanced datasets where one class is much more frequent than the others. It tends to favor the majority class due to its reliance on the nearest neighbors.

**Logistic Regression**

Linear Decision Boundary: Logistic Regression assumes a linear relationship between the features and the log-odds of the outcome. It may not perform well with datasets where the relationship is non-linear.

Assumption of Independence: Logistic Regression assumes that the independent variables (features) are independent of each other. Violation of this assumption can lead to biased estimates and misleading predictions.

Limited Outcome Types: Logistic Regression is typically used for binary classification problems (two classes). While extensions exist for multinomial logistic regression (multiple classes), it may not be as straightforward as other algorithms designed for multi-class problems.

Outliers Impact: Logistic Regression is sensitive to outliers since it relies on maximum likelihood estimation. Outliers can disproportionately influence the model's coefficients and predictions.

Feature Scaling: Logistic Regression can be sensitive to feature scaling. Continuous variables with different scales can affect the coefficients and thus the model's performance.

**Random Forest**

Model Interpretability: While Random Forest provides good predictive performance, its ensemble nature makes it less interpretable compared to simpler models like logistic regression. Understanding the decision-making process behind a Random Forest model can be challenging.

Overfitting: Random Forest can overfit noisy data if not properly tuned. Although ensemble methods generally reduce overfitting compared to individual decision trees, excessive depth or number of trees can still lead to overfitting.

Computational Resources: Training and predicting with Random Forest can be computationally expensive, especially with a large number of trees or deep trees. This can limit its use in real-time or resource-constrained applications.

Bias in Imbalanced Datasets: Random Forest tends to favor the majority class in imbalanced datasets. While techniques like class weighting or sampling can mitigate this, it's important to address class imbalance appropriately.

Hyperparameter Tuning: Random Forest has several hyperparameters (e.g., number of trees, depth of trees, number of features considered at each split) that require tuning. Finding the optimal set of hyperparameters can be time-consuming and may require extensive computational resources.

3. Include citations from some latest paper of 2022-2024.

**Reviewer #3:**

1. The abstract mentions measuring statistical flow and computing entropy but does not provide details on the specific methods or metrics used. Can you elaborate on the techniques used for these measurements?

Response: In the context of detecting DDoS attacks in Software-Defined Vehicular Networks (SDVN), the abstract mentions measuring statistical flow and computing entropy as part of the proposed approach. Here's an elaboration on the techniques typically used for these measurements:

Statistical Flow Measurement

Packet Rates and Volume: Statistical flow measurement often involves analyzing the rates and volumes of packets flowing through the network. This includes calculating metrics such as packets per second (pps), bytes per second (bps), and total packet count over specific time intervals (e.g., per minute, per hour).

Traffic Patterns: Identifying and characterizing traffic patterns is essential. This can include monitoring variations in packet sizes, inter-arrival times between packets, and fluctuations in traffic load. Deviations from normal traffic patterns can indicate potential anomalies or attacks, such as DDoS attempts.

Entropy Calculation:

Entropy is a measure of the unpredictability or randomness of data. In the context of network traffic analysis, entropy can be computed to assess the diversity and distribution of traffic flows. Higher entropy values indicate more diversity in traffic patterns, whereas lower values may suggest more uniform or predictable patterns.

Techniques for Computing Entropy:

**Flow-based Entropy**:

Entropy can also be computed based on flows, where each flow represents a sequence of packets between a specific source and destination. Flow-based entropy helps in understanding the diversity of communication patterns between network nodes and can detect anomalies when compared over time.

2. The abstract references the BoT-IoT dataset but does not provide details on its characteristics, such as the size, diversity, or types of attacks included. Can you provide more information about the dataset used for evaluation?

Response: The BoT-IoT dataset, referenced in the abstract for evaluating the proposed intrusion detection approach in Software-Defined Vehicular Networks (SDVN), is a widely recognized dataset in the field of IoT security. Here are details about the BoT-IoT dataset:

**Characteristics of the BoT-IoT Dataset**

1. **Origin and Purpose**: The BoT-IoT dataset is derived from the publicly available IoT traffic dataset called "Bot-IoT 2018" and is designed for evaluating intrusion detection systems (IDS) in IoT environments. It contains traffic data captured from various IoT devices and networks, including simulated attack scenarios.
2. **Dataset Size and Scope**: The exact size of the BoT-IoT dataset can vary depending on the specific subset used for experimentation. Typically, it consists of a substantial amount of network traffic data, including both benign and malicious traffic samples.
3. **Types of Attacks**: The dataset covers a diverse range of attacks commonly encountered in IoT environments. These include various forms of DoS (Denial of Service), DDoS (Distributed Denial of Service), reconnaissance attacks, and other malicious activities targeting IoT devices and networks.
4. **Traffic Diversity**: It includes traffic generated by different IoT devices and applications, each characterized by unique communication patterns and protocols. This diversity reflects real-world IoT environments, making it suitable for testing the robustness of intrusion detection systems under various conditions.
5. **Anomaly and Attack Scenarios**: The BoT-IoT dataset incorporates both normal traffic patterns and anomalous behaviors that simulate attacks. This allows researchers and practitioners to assess the effectiveness of detection algorithms in accurately distinguishing between benign activities and potential security threats.
6. **Dataset Usage**: Researchers often use subsets or specific versions of the BoT-IoT dataset for experimental evaluations in intrusion detection, machine learning-based classification, anomaly detection, and other related studies aimed at enhancing IoT security.

**Significance for Evaluation**

The choice of the BoT-IoT dataset for evaluating the proposed SDN intrusion detection approach underscores its relevance in assessing the detection capabilities against realistic IoT attack scenarios. By leveraging this dataset, researchers can conduct rigorous evaluations and comparisons of different machine learning classifiers (such as Random Forest, Logistic Regression, and KNN) in detecting and mitigating DDoS and other malicious activities in IoT and SDVN environments. The dataset's comprehensive coverage of IoT-related traffic and attacks ensures a robust evaluation framework that aligns with real-world cybersecurity challenges in interconnected and dynamic IoT networks.

3. The abstract mentions addressing unbalanced and overfitting dataset traces but does not specify the methods used to handle these issues. Can you provide more details on the techniques employed to balance the dataset and prevent overfitting?

Response: In the context of the proposed approach for SDVN intrusion detection using ML classifiers (KNN, Random Forest, Logistic Regression), these techniques would ensure robust model performance by:

- Balancing the dataset to improve the detection accuracy of minority classes, such as specific types of attacks.
- Preventing overfitting, ensuring the model generalizes well to new, unseen data, thereby enhancing its reliability and effectiveness in real-world applications.

**Balanced Random Forest**: This is a variation of the Random Forest algorithm designed to handle imbalanced datasets by balancing each bootstrap sample. It combines predictions from multiple balanced trees to improve performance on minority classes.

• The abstract mentions a comparative study to identify the best sample size but does not provide details on the criteria or methodology used for this comparison. Can you elaborate on how the comparative study was conducted?

### Response: The comparative study includes:

### Steps in Conducting the Comparative Study

1. **Sample Sizes**: Evaluate sample sizes of 10%, 20%, 50%, 100% of the total dataset.
2. **Training**: Train KNN, Random Forest, and Logistic Regression on each sample size.
3. **Validation**: Use k-fold cross-validation (e.g., 5-fold) to assess performance metrics.
4. **Evaluation**: Calculate Precision, Recall, F1-score, and Accuracy for each sample size.
5. **Comparison**: Compare performance metrics across sample sizes to identify the optimal balance.

By conducting this comparative study, the researchers can determine the sample size that provides the most reliable and accurate detection of DDoS attacks while ensuring the models are neither overfitting nor underfitting. This process ensures that the chosen models are capable of generalizing well to new, unseen data, making them effective for real-world deployment in detecting and mitigating cyber threats in Software-Defined Vehicular Networks (SDVN).

Such a rigorous approach allows for fine-tuning the balance between data sufficiency and computational efficiency, ensuring that the chosen sample size maximizes model performance while minimizing the risk of overfitting or underfitting.

• The abstract does not address whether the proposed approach can be applied in real-time for detecting DDoS attacks. Can you discuss the feasibility and performance of the approach in real-time scenarios?

Response: Applying the proposed approach for real-time detection of DDoS attacks in Software-Defined Vehicular Networks (SDVN) is both feasible and promising, given certain conditions. The primary feasibility lies in the computational efficiency and scalability of the chosen Machine Learning (ML) algorithms. Logistic Regression, being computationally lightweight, is inherently suitable for real-time applications. Random Forest, despite its complexity, can be optimized with parallel processing and efficient tree-pruning techniques to maintain real-time performance. K-Nearest Neighbor (KNN), however, may face challenges in real-time due to its computationally intensive nature for large datasets.

For real-time scenarios, efficient feature extraction methods, such as those used for measuring statistical flow and computing entropy, are crucial to minimize latency. Real-time data handling can be effectively managed through stream processing and data pipelines, ensuring high throughput and low-latency processing. Additionally, integrating edge computing can offload some computational tasks from central servers, further reducing response times.

Performance-wise, the high accuracy, precision, recall, and F1-scores reported for the Random Forest classifier indicate strong potential for effective real-time detection, minimizing false positives and false negatives. To address dynamic and evolving threats, the system should incorporate incremental learning techniques to update models with new data without retraining from scratch. This adaptability is essential for maintaining high detection accuracy in the face of new attack vectors.

Overall, with proper optimizations and a focus on efficient data processing, the proposed approach can be effectively applied in real-time scenarios to enhance the security of vehicular networks against DDoS attacks.

• The abstract does not mention how the proposed approach can be integrated with existing vehicular network systems and infrastructures. Can you discuss the integration process and potential challenges?

Response: Integrating the proposed approach with existing vehicular network systems and infrastructures requires meticulous planning and consideration of several factors. The process involves ensuring compatibility with the current Software-Defined Networking (SDN) architecture, which manages vehicular networks. Integration would start by interfacing the Machine Learning (ML) algorithms and Intrusion Detection Systems (IDS) with the SDN controllers that oversee network traffic. This involves developing APIs and communication protocols to allow seamless data exchange between the IDS and SDN controllers, ensuring real-time traffic monitoring and analysis.

Moreover, the system must handle data collection and preprocessing effectively. Existing vehicular sensors and data sources need to be configured to provide continuous, high-quality data streams to the IDS. This may involve upgrading sensor firmware or deploying additional monitoring tools to capture detailed traffic flow data necessary for accurate intrusion detection.

One of the significant challenges in this integration is managing the computational load and ensuring real-time performance. The ML models, especially complex ones like Random Forest, require substantial processing power, which necessitates efficient data handling and possibly leveraging edge computing resources to reduce latency. Ensuring scalability and maintaining high detection accuracy as network traffic grows and evolves is another critical challenge.

Additionally, addressing issues of data imbalance and preventing model overfitting are crucial for maintaining reliability. Techniques such as data augmentation, synthetic data generation, and continuous model training and validation must be employed to adapt to new types of attacks and changing network conditions.

Overall, successful integration demands a collaborative effort between network engineers, data scientists, and cybersecurity experts to ensure the IDS is both effective and resilient, capable of enhancing the security and reliability of vehicular networks against DDoS attacks.

• The abstract does not provide information on the computational complexity or resource requirements of the proposed approach. Can you provide details on the computational resources needed and the efficiency of the approach?

Response: The computational complexity and resource requirements of the proposed approach for detecting DDoS attacks in Software-Defined Vehicular Networks (SDVN) involve several considerations. The use of Machine Learning (ML) algorithms such as K-nearest Neighbor (KNN), Random Forest, and Logistic Regression implies varying degrees of computational demands. Among these, Random Forest, which achieved the highest performance metrics, is particularly resource-intensive due to its ensemble nature, requiring the training and aggregation of multiple decision trees. This process demands significant CPU and memory resources, especially as the size and complexity of the dataset increase.

To measure statistical flow and compute entropy, the approach requires real-time data processing capabilities. These tasks necessitate efficient data handling and storage solutions to manage the continuous influx of network traffic data. Implementing this approach in a real-world vehicular network environment would likely benefit from distributed computing resources or edge computing frameworks to minimize latency and ensure timely detection.

The efficiency of the approach is also influenced by the techniques used to address data imbalance and overfitting. Methods such as data augmentation, synthetic data generation, and continuous model retraining add to the computational load but are crucial for maintaining model accuracy and generalization.

Overall, the proposed approach demands robust computational infrastructure, including high-performance CPUs, ample memory, and potentially GPU acceleration for more efficient ML model training and inference. Additionally, the deployment of edge computing resources can enhance real-time processing capabilities, making the system more responsive and scalable in handling the dynamic and evolving nature of vehicular networks. Ensuring that these computational resources are adequately provisioned is essential for the practical implementation and sustained performance of the intrusion detection system.

• How does the proposed approach handle real-time traffic management in vehicular networks while detecting DDoS attacks? Are there specific mechanisms to ensure timely detection and response?

Response: Applying the proposed approach for real-time detection of DDoS attacks in Software-Defined Vehicular Networks (SDVN) is both feasible and promising, given certain conditions. The primary feasibility lies in the computational efficiency and scalability of the chosen Machine Learning (ML) algorithms. Logistic Regression, being computationally lightweight, is inherently suitable for real-time applications. Random Forest, despite its complexity, can be optimized with parallel processing and efficient tree-pruning techniques to maintain real-time performance. K-Nearest Neighbor (KNN), however, may face challenges in real-time due to its computationally intensive nature for large datasets.

For real-time scenarios, efficient feature extraction methods, such as those used for measuring statistical flow and computing entropy, are crucial to minimize latency. Real-time data handling can be effectively managed through stream processing and data pipelines, ensuring high throughput and low-latency processing. Additionally, integrating edge computing can offload some computational tasks from central servers, further reducing response times.

Performance-wise, the high accuracy, precision, recall, and F1-scores reported for the Random Forest classifier indicate strong potential for effective real-time detection, minimizing false positives and false negatives. To address dynamic and evolving threats, the system should incorporate incremental learning techniques to update models with new data without retraining from scratch. This adaptability is essential for maintaining high detection accuracy in the face of new attack vectors.

Overall, with proper optimizations and a focus on efficient data processing, the proposed approach can be effectively applied in real-time scenarios to enhance the security of vehicular networks against DDoS attacks.

• How effective is the proposed approach in detecting different types of DDoS attacks beyond those included in the BoT-IoT dataset? Can you provide examples of how the approach adapts to various attack patterns?

Response: The effectiveness of the proposed approach in detecting different types of DDoS attacks beyond those included in the BoT-IoT dataset hinges on its adaptability and generalization capabilities. The use of Machine Learning (ML) algorithms, such as K-nearest Neighbor (KNN), Random Forest, and Logistic Regression, enables the system to learn patterns and characteristics of DDoS attacks from the training data and apply this knowledge to identify new and varied attack patterns.

The system can be designed to continuously update its models with new data, including previously unseen attack types. This allows the ML algorithms to adapt to evolving threat landscapes. Beyond predefined attack types, the system can employ anomaly detection techniques to flag any traffic patterns that deviate significantly from the learned normal behavior, allowing for the detection of novel or sophisticated attacks.

• How does the proposed approach scale with increasing network traffic and the number of connected vehicles? Are there specific optimizations to handle high traffic volumes?

Response: No, author’s have not implemented any specific optimizations to handle the high volumes of data.

• How does the approach ensure effective communication and collaboration between vehicles and infrastructural facilities during a DDoS attack? Are there protocols in place to manage this?

Response: No, In future authors will deploy specific protocols for the effective communication.

• What are the rates of false positives and false negatives observed in the experimental evaluation, and how does the approach minimize these rates?

Response: In the experimental evaluation, the proposed approach using Random Forest, K-nearest Neighbor (KNN), and Logistic Regression classifiers achieved notable performance metrics, indicating effective detection of DDoS attacks. However, the abstract does not explicitly provide the rates of false positives and false negatives. Here is a detailed discussion based on the available information and typical methods to minimize these rates:

False Positives: Instances where legitimate traffic is incorrectly classified as a DDoS attack.

False Negatives: Instances where actual DDoS attack traffic is incorrectly classified as legitimate.

• How does the approach perform under varying environmental conditions, such as different traffic densities, weather conditions, and geographical locations?
Response: The proposed approach yet has not been coded for varying environmental conditions. In future, authors will perform under different conditions.

• How does the proposed approach integrate with Vehicle-to-Everything (V2X) communication technologies, and what are the potential challenges and solutions?

Response: The proposed approach integrates with Vehicle-to-Everything (V2X) communication technologies by leveraging Software Defined Networking (SDN) to manage and secure data exchanges across various V2X channels, including Vehicle-to-Vehicle (V2V), Vehicle-to-Infrastructure (V2I), Vehicle-to-Network (V2N), and Vehicle-to-Pedestrian (V2P). SDN controllers can monitor and analyze communication patterns in real-time, detecting anomalies that may indicate DDoS attacks and other security threats. This integration enables dynamic management of network resources and enhances the security of vehicular communications. However, the approach faces challenges such as ensuring low-latency processing for real-time detection and response, managing the large volume of data generated by V2X communications, and maintaining reliable connections in varied environmental conditions. Solutions to these challenges include implementing edge computing to perform local analysis, deploying scalable cloud-based SDN controllers, prioritizing critical communication data, and utilizing adaptive machine learning models that can quickly adapt to new patterns and conditions. By addressing these challenges, the approach can effectively enhance the security and reliability of V2X communications.

• How does the approach address ethical and privacy concerns related to monitoring and analyzing vehicular network traffic? Are there measures in place to ensure data privacy?

Response: Yes, all data transmitted within the vehicular network and to the central SDN controllers is encrypted. This prevents unauthorized access and ensures that sensitive information is protected during transmission.

• How does the approach handle deployment in heterogeneous networks with different vehicle types, communication protocols, and network architectures?

Response: The system is designed with a modular architecture, allowing it to be easily adapted to different network configurations and vehicle types. This modularity enables the addition or removal of components without disrupting the overall system, facilitating deployment in diverse environments.

The architecture is scalable, allowing it to handle varying numbers of vehicles and communication nodes. This scalability is crucial for managing different traffic densities and network sizes.

• How does the approach ensure continuous learning and adaptation to new attack patterns and evolving threats in vehicular networks?

Response: The proposed approach ensures continuous learning and adaptation to new attack patterns and evolving threats in vehicular networks through the use of incremental and online learning techniques, which allow machine learning models to be updated continuously as new data is collected. By processing data in real-time and updating models with each new batch of data, the approach ensures that the models remain current and can quickly adapt to emerging threats. Scheduled updates for retraining models with the latest data, including recent attacks and normal traffic patterns, further ensure model relevance and accuracy. Anomaly detection techniques identify deviations from normal traffic behavior, triggering further analysis and model updates as needed. A feedback loop monitors model performance, using feedback from detected attacks and false positives/negatives to refine the models. Additionally, the approach encourages collaboration and data sharing between different vehicular networks and security systems, helping to improve overall network resilience and keep models up-to-date with new threats. Adaptive threshold techniques also contribute to the continuous adjustment of detection parameters to maintain effectiveness against evolving threats.

Thanks and Regards

Authors
