# Supplementary material for: Effective DDoS attack detection in software-defined vehicular networks using statistical flow analysis and machine learning
Source: PLoS One. 2024 Dec 18;19(12):e0314695. doi: 10.1371/journal.pone.0314695 (PMC11654938; doi:10.1371/journal.pone.0314695)
Supplement: Attachment — Submitted filename: minor revision PLOS ONE.docx [file pone.0314695.s002.docx]

**Point-wise Detailed Response to Editor and Reviewers’ comments**

Title: Effective DDoS Attack Detection in Software-Defined Vehicular Networks Using Statistical Flow Analysis and Machine Learning

Authors: Himanshi Babbar, Shalli Rani, Maha Driss

**Dear Editors and Reviewers:**

We are thankful to you for spending your valuable time for making a review and for constructing the comments on our manuscript. These comments are valuable and very helpful for revising and improving our paper. We have studied comments carefully and have made correction as marked in the revised manuscript. We have tried our best to address the mentioned comments to revise our manuscript in the hope that these revisions will meet your requirement. The following changes have been made in the manuscript as per the received comments.

*The changes made in the manuscript as per received comments have been highlighted in blue color.

**Editor’s Comments**

1. Please check carefully for any non compliance issue between the metrics mentioned in the abstract, introduction and the conclusion parts (your models accuracy : 91% in the abstract ; 96.3% (introduction; line 112 and conclusion; line 713).

Response: Sorry for the non-compliance. The manuscript has been thoroughly read and made corrections as per the suggestion. The changes will be reflected with blue color on page no 5 in main contributions 6 point, and conclusion section.

2. Please insert a paragraph at the end of the introduction to highlight the structure of the manuscript.

Response: The organization of the paper has been inserted on page 5 and it is highlighted with blue color.

3. Please carefully review the manuscript and ensure that acronyms are explained only during their first use.

Response: The overall manuscript has been thoroughly read and ensured that acronyms are used only once.

Comments to the Author

**Comments to the Author**

1. If the authors have adequately addressed your comments raised in a previous round of review and you feel that this manuscript is now acceptable for publication, you may indicate that here to bypass the “Comments to the Author” section, enter your conflict of interest statement in the “Confidential to Editor” section, and submit your "Accept" recommendation.

Reviewer #2: All comments have been addressed

Response: Thank you for the feedback.

Reviewer #4: All comments have been addressed

Response: Thank you for the feedback.

2. Is the manuscript technically sound, and do the data support the conclusions?

The manuscript must describe a technically sound piece of scientific research with data that supports the conclusions. Experiments must have been conducted rigorously, with appropriate controls, replication, and sample sizes. The conclusions must be drawn appropriately based on the data presented.

Reviewer #2: Yes

Response: Thank you for the feedback.

Reviewer #4: Yes

Response: Thank you for the feedback.

3. Has the statistical analysis been performed appropriately and rigorously?

Reviewer #2: Yes

Response: Thank you for the feedback.

Reviewer #4: Yes

Response: Thank you for the feedback.

4. Have the authors made all data underlying the findings in their manuscript fully available?

The [PLOS Data policy](http://track.editorialmanager.com/CL0/http:%2F%2Fwww.plosone.org%2Fstatic%2Fpolicies.action%23sharing/1/010f01931986f12c-8b6839dc-7cc8-4d59-a8cf-279a92defc8e-000000/aUDO3kKU0VChmwqT2AFYSLYmRa62z2MzeTNQZueuDVQ=184) requires authors to make all data underlying the findings described in their manuscript fully available without restriction, with rare exception (please refer to the Data Availability Statement in the manuscript PDF file). The data should be provided as part of the manuscript or its supporting information, or deposited to a public repository. For example, in addition to summary statistics, the data points behind means, medians and variance measures should be available. If there are restrictions on publicly sharing data—e.g. participant privacy or use of data from a third party—those must be specified.

Reviewer #2: Yes

Response: Thank you for the feedback.

5. Is the manuscript presented in an intelligible fashion and written in standard English?

PLOS ONE does not copyedit accepted manuscripts, so the language in submitted articles must be clear, correct, and unambiguous. Any typographical or grammatical errors should be corrected at revision, so please note any specific errors here.

Reviewer #2: Yes

Reviewer #4: Yes

6. Review Comments to the Author

Please use the space provided to explain your answers to the questions above. You may also include additional comments for the author, including concerns about dual publication, research ethics, or publication ethics. (Please upload your review as an attachment if it exceeds 20,000 characters)

Reviewer #2: Thank you very much for following my comments point to point. You can include some directions in the manuscript for future research.

Response: The future scope has been included in the conclusion section on page no 24 and it is highlighted with blue color.

Reviewer #4: This paper presents an intriguing research direction and makes a valuable contribution to the field.

- Comprehensive Evaluation: The paper offers a thorough analysis of the proposed method, demonstrating a solid understanding of the topic.
- High Accuracy: The results indicate a high level of accuracy.
- Methodical Process: The research follows a clear and methodical approach.
- Addressing Real-World Challenges: The study is relevant to practical applications.
- The references are up-to-date and relevant.

Response: Thank you for the feedback.

**Thanks and Regards**

**Authors**
